# Long-standing Sporadic Pancreatic Insulinoma: Report of a Rare Case

**DOI:** 10.7759/cureus.6947

**Published:** 2020-02-11

**Authors:** Zainab Majid, Faryal Tahir, Syed Ali Haider

**Affiliations:** 1 Internal Medicine, Dow University of Health Sciences, Karachi, PAK; 2 Surgery, Dow University of Health Sciences, Karachi, PAK

**Keywords:** insulinoma, hypoglycemia, pancreas, neuroendocrine tumor, endocrine carcinoma, surgery, men 1 syndrome, pancreatic cancer

## Abstract

Insulinomas are rare, functional pancreatic neuroendocrine tumors arising from the pancreatic multipotent stem cells or neuroendocrine islet, occurring with a higher proportion in females. Majority of insulinomas have a sporadic etiology; however, only 5%-10% develop as a part of multiple endocrine neoplasm type 1 syndrome. They usually present with symptoms of hypoglycemia including disturbance in orientation, tremors, diaphoresis, altered mental state, seizures and visual changes among others. The diagnosis is based on appreciation of the classic Whipple triad, i.e. neuroglycopenic symptoms and sympathetic drive along with low serum glucose levels (<50 mg/dL) and a complete reversibility of these symptoms with prompt administration of glucose. The gold standard treatment for insulinoma involves complete surgical excision (i.e. enucleation), which is curative in 90% of the patients. Health care physicians should have a high index of suspicion for this tumor in patients presenting with neurological and sympathetic symptoms, particularly if they are resolved after eating. Here, we report the case of a 48-year-old female with the history of multiple episodes of hypoglycemic symptoms for the past two years which improved on glucose intake. Furthermore, we also summarized the discussion regarding diagnosis and management of pancreatic insulinoma.

## Introduction

Insulinomas are rare, functional pancreatic neuroendocrine tumors (NETs) arising from the pancreatic multipotent stem cells or neuroendocrine islet cells with per annum cases of one to three for every million people [1]. Insulinomas tend to have a higher predilection for females and are usually diagnosed around 30-60 years of age [2]. Majority of insuliomas have a sporadic etiology; however, only 5%-10% develop as a part of multiple endocrine neoplasia type 1 (MEN1) syndrome and tend to be multiple and even malignant in 25% of the cases [3]. Approximately 80% of insuliomas have dimensions of less than 2 cm and thus, pose diagnostic and imaging challenges [4]. Clinically, the presence of Whipple’s triad is indicative of insulinoma. The symptoms of hypoglycemia accompany disturbance in orientation, tremors, diaphoresis, altered mental state, seizures and visual changes [5]. Since these tumors are commonly found as solitary, small in size and benign, a vast majority (90%) of the patients can be cured via complete surgical resection of the tumor, i.e. enucleation, being the treatment of choice [6].

We report the case of a 48-year-old female with a history of multiple episodes of hypoglycemic symptoms for the past two years, which improved on eating or glucose intake.

## Case presentation

A 48-year-old female with no known comorbidities presented to the medical outpatient department of Civil Hospital Karachi with complaint of recurrent episodes of altered sensorium, lethargy, drowsiness, sweating, blurring of vision and palpitations with no loss of consciousness for the past two years. The patient highlighted that all these symptoms improved on eating, particularly with sugar intake. She also complained of sudden unintentional weight gain which was undocumented. The frequency of these episodes had increased gradually over the past two years for which she was being managed conservatively with intravenous (IV) administration of dextrose at a local health care set up. The patient also had a significant family history of diabetes mellitus (DM). The patient was admitted to the hospital for observation and further investigation.

On admission, the patient was afebrile but lethargic and drowsy, with a blood pressure of 140/90 mmHg, heart rate of 80 beats/min and a respirator rate of 16 breaths/min. On examination, the abdomen was soft and non-tender with audible gut sounds. Fullness in the epigastric region was appreciated with no organomegaly. Pulmonary, cardiac and neurological examinations were unremarkable. Routine hematological and biochemical investigations were within normal ranges.

Shortly after hospitalization, a 72-hour supervised fasting test was performed during which the patient developed hypoglycemic symptoms which were promptly managed by administration of IV dextrose 25% with water. The subsequent investigations revealed low blood glucose levels along with elevated levels of serum insulin and connecting peptide (C-peptide). Exact values are summarized in Table 1.

**Table 1:** Laboratory investigations during 72-hour supervised fasting test RBS, random blood sugar; C-peptide, connecting peptide

Feature	Patient’s Value	Reference Range
Insulin (µIU/mL)	31.8 ↑	2.6-24.9
RBS (mg/dL)	35 ↓	70-110
Serum C-peptide (ng/mL)	3.53 ↑	0.5-2.0

**Figure 1:**
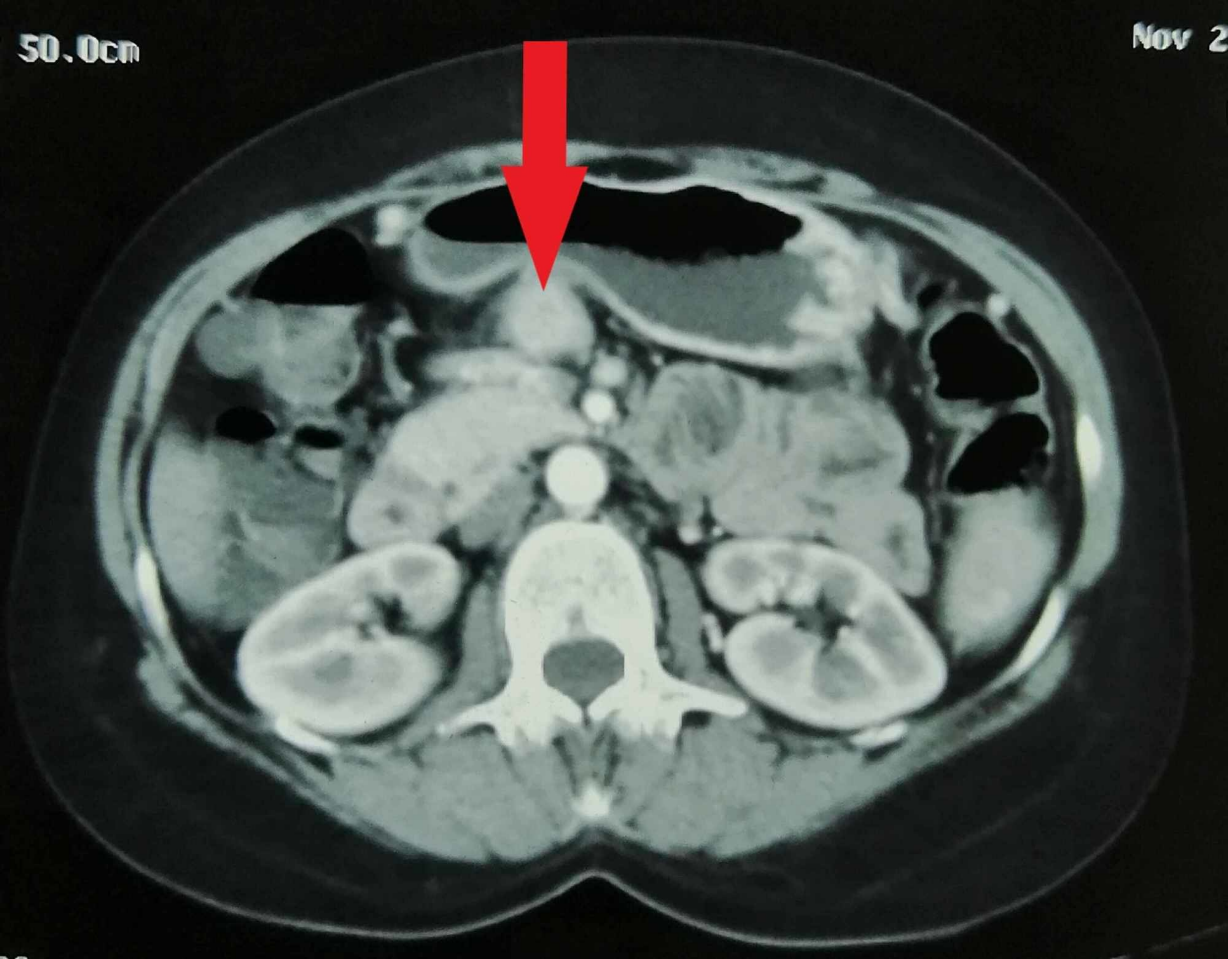
Axial view of abdominal CECT showing a hyperdense, ill-defined mass arising from the pancreatic body CECT, contrast-enhanced computed tomography

**Figure 2:**
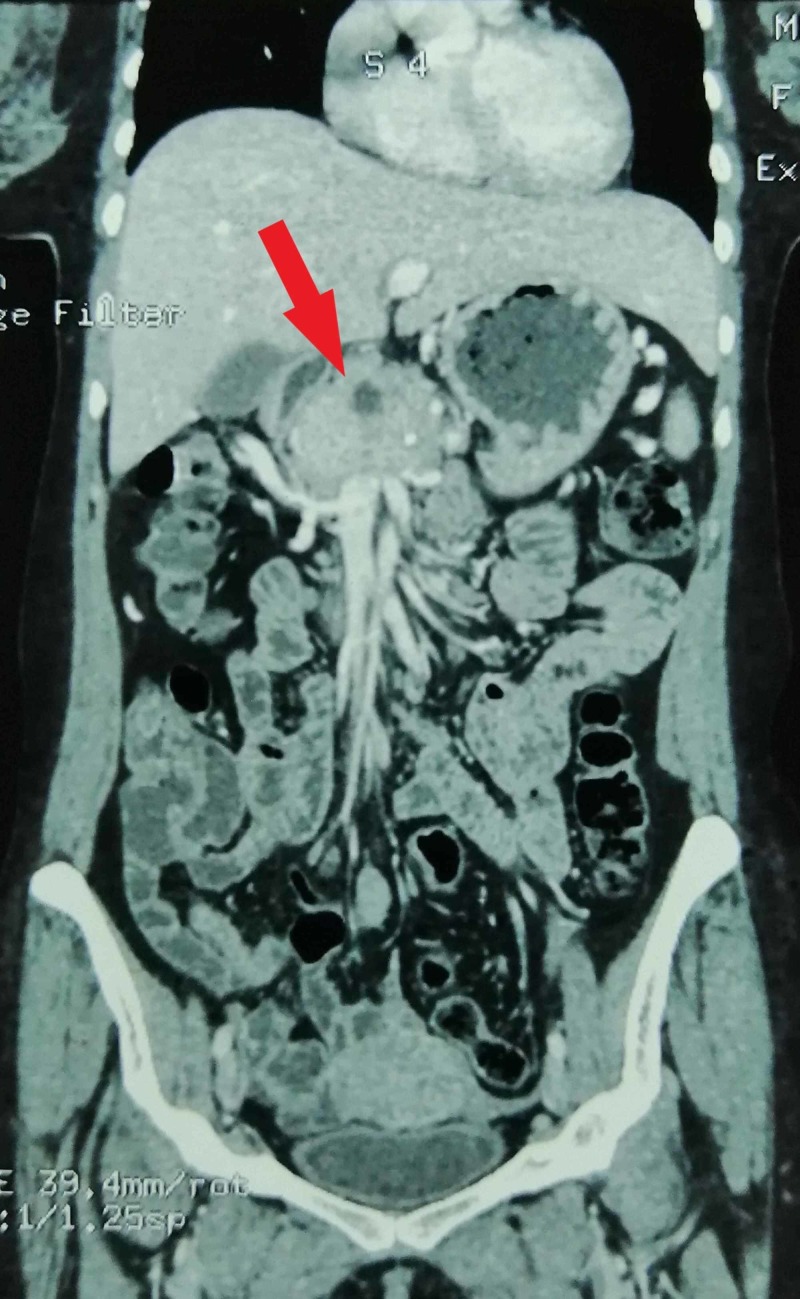
Coronal view of abdominal CECT showing a hyperdense, ill-defined mass arising from the pancreatic body CECT, contrast-enhanced computed tomography

After stabilization of the patient, a transabdominal ultrasound (TUS) was performed. It revealed a lobulated, hyperechoic mass in the body of the pancreas. A provisional diagnosis of a pancreatic mass was made and pancreatic insulinoma was included as a differential. It was then followed by a contrast-enhanced computed tomography (CT) of abdomen which demonstrated an ill-defined, avidly enhancing lesion in the body of the pancreas measuring 3 x 4.5 cm with internal areas of necrosis and intact fat planes. The mass was found abutting the adjacent vessels posteriorly and greater curvature of the stomach anteriorly (Figures 1, 2).

Furthermore, hormonal studies were performed which showed serum cortisol, thyroid profile, parathormone and adrenocorticotropic hormone profile within normal ranges. Hence, the possibility of MEN1 was excluded and the patient was referred to the surgical unit for further management, with the clinical and radiological impression of solitary, benign pancreatic insulinoma.

After assessing the patient for surgical fitness, a complete surgical resection of insulinoma was done with no intraoperative evidence of invasion or liver metastasis. Pathological examination of the resected specimen revealed positive staining for synaptophysin and hence, functioning pancreatic insulinoma was confirmed. Postoperatively, the patient was kept under observation for five days in which time she exhibited no signs of hypoglycemia or any other surgical complications. Blood sugar and insulin levels improved significantly, and the patient was discharged with an uneventful recovery with the advice to follow-up.

## Discussion

Hypoglycemia was first recognized in the early 19th century. After the invention of insulin and its implementation in the treatment of diabetic patients, in 1920s, non-diabetic hypoglycemia was suspected to be a consequence of hyperinsulinism. In 1924, Seale Harris published a description highlighting the same association. The first case of hypoglycemia that was surgically cured by removing an islet cell tumor was reported in 1929. 

Normally, the insulin is secreted in response to higher blood glucose levels which then acts to decrease serum glucose concentrations back to normal levels, i.e. a point where further insulin is not required and ultimately, as a negative feedback mechanism, insulin levels drop. Insulinoma is a beta-cell-derived NET of pancreas which is responsible for unregulated secretion of insulin. Most insulinomas (99%) arise from the pancreas and tend to be solitary, small (less than 2 cm) and benign, i.e. limited to their origin, but a few metastasize. In some cases (5%), they occur as multiple and malignant neoplasms, often in association with MEN1. MEN1 is an autosomal dominant disorder characterized by parathyroid hyperplasia, pituitary adenoma (anterior pituitary) and NET of pancreas or duodenum as a consequence of mutation in the MEN1 gene on chromosome 11. In comparison with solitary insulinomas, MEN1 associated insulinomas are earlier to develop and often multicentric [5].

Patients with insulinoma usually suffer from neuroglycopenic symptoms (e.g. confusion, behavioral changes, loss of orientation) as a consequence of chronic insufficient supply of glucose to the brain which ultimately causes deterioration in neuronal function. As a result, the patient experiences recurrent attacks of headache, lethargy, blurred vision and diplopia, more often following physical activity or fasting. Profound hypoglycemia can cause seizures, coma, and in severe cases, permanent neurological damage. Catecholaminergic response to hypoglycemia can further produce symptoms of tachycardia, palpitations, hyperhidrosis, hunger and anxiety. Some patients may also complain of sudden weight gain, as evident by our case. Therefore, the diagnosis is based on appreciation of the classic Whipple triad, i.e. neuroglycopenic symptoms and sympathetic drive along with low serum glucose levels (<50 mg/dL) and a complete reversibility of these symptoms with prompt administration of glucose [7]. It is evident from our case where all the components of this triad were acknowledged.

Insulinoma is usually suspected in an individual with symptomatic fasting hypoglycemia, as in our case. Nevertheless, pancreatic insulinoma remains a diagnostic dilemma as it is typically diagnosed after less than 1.5 years following the onset of symptoms [8]. In some cases, patients may be misdiagnosed as neurological or psychiatric disorders, and may undergo unnecessary imaging with unprofitable psychiatric treatment.

Clinically, a 72-hour supervised fasting test is the definitive diagnostic modality for insulinoma, in which despite hypoglycemia and absence of serum sulfonylurea, an inappropriate rise in the levels of insulin (≥6 μU/mL) and C-peptide (≥0.2 nmol/L) is appreciated [8]. Upon the same investigation, our patient also showed elevated serum levels of insulin and C-peptide. After establishing the diagnosis, different imaging techniques might be opted to localize the tumor. This is vital for planning the surgery because not all tumors are palpable intraoperatively. Non-invasive imaging modalities include TUS, CT scan, magnetic resonance imaging, pentetreotide scintigraphy, and positron emission tomography. Despite all these techniques, the investigation must be tailored according to the availability and local radiological skills [5]. In our setup, TUS was initially carried out which showed suspicious findings. It was then followed by a CT scan to confirm the presence, location and stage of the tumor. In cases of negative imaging results, invasive techniques can be opted which include endoscopic ultrasound (EUS), selective arterial calcium stimulation test (SACST) and angiographic pancreatic vein catheterization (to sample the blood for detecting insulin levels). Depending on the location of insulinoma, an EUS exhibits a sensitivity of 40%-93% for detection of the tumor [9].

The gold standard treatment for insulinoma involves complete surgical excision (i.e. enucleation) as most cases exhibit a solitary and benign tumor. For more extensive tumors, a part of the pancreas can also be removed via procedures named as distal pancreatectomy and Whipple procedure. Apart from open surgeries, laparoscopic exploration with intraoperative ultrasound (IOUS) is also being considered nowadays. In a prospective study, Grover et al. called IOUS to be equivalent to SACST and venous sampling for the localization of insulinoma [10]. For those unfit for surgery or when tumor resection is unsuccessful, medications such as diazoxide and somatostatin can be used to inhibit the release of insulin. In the advanced metastatic disease, a combination chemotherapy using doxorubicin (fluorouracil if doxorubicin is contraindicated) and streptozotocin can be considered.

Surgical resection of insulinoma has been regarded as satisfactory, with no mortality and good functional results [11]. Following surgical excision, a vast majority (90%-95%) of patients with benign insulinomas can expect long-term cure with complete resolution of preoperative symptoms. Recurrence rate is minimal (3%) [12]. In patients with multiple tumors, the surgery is followed by persistent or recurrent hypoglycemia. DM may occur in approximately 2% of patients, mainly in those who undergo major pancreatic resections. Up to 14 years post-resection, a non-functioning metastatic disease to the liver may develop [12]. For this metastatic growth, hepatic arterial embolization or occlusion can be used.

## Conclusions

Pancreatic insulinoma being a very rare tumor should not be overlooked. Health care physicians should have a high index of suspicion for this tumor in patients presenting with neurological and sympathetic symptoms, particularly if they are resolved after eating. Investigation and treatment should be tailored according to the availability of techniques and status of the patient, respectively. A multidisciplinary approach involving medical and surgical team along with oncologists and pathologists is crucial for timely diagnosis and improved outcomes.
